# Comparing Ocular Responses to Caloric Irrigation and Electrical Vestibular Stimulation in Vestibular Schwannoma

**DOI:** 10.3389/fneur.2019.01181

**Published:** 2019-11-08

**Authors:** Stuart W. Mackenzie, Richard Iriving, Peter Monksfield, Attila Dezso, Nicholas Dawe, Karen Lindley, Raymond F. Reynolds

**Affiliations:** ^1^School of Sport, Exercise and Rehabilitation Sciences, University of Birmingham, Birmingham, United Kingdom; ^2^Centre for Rare Diseases, University Hospital Birmingham, Birmingham, United Kingdom

**Keywords:** electrical vestibular stimulation, caloric irrigation, vestibular schwannoma, asymmetry ratio, ocular torsion

## Abstract

Electrical Vestibular Stimulation (EVS) is a non-invasive technique for activating the vestibular-ocular reflex, evoking mainly a torsional eye movement response. We have previously demonstrated that this response can be used to detect vestibular asymmetry in patients with vestibular schwannoma (VS). Here we perform a direct comparison of EVS with caloric irrigation in this patient group. We studied 30 patients with unilateral VS, alongside an equal number of aged-matched healthy control subjects. EVS current was delivered to the mastoid process in a monaural configuration using a sinusoidal stimulus (2 Hz; ± 2 mA; 10 s), with an electrode placed over the spinous C7 process. Evoked eye movements were recorded from the right eye in darkness using an infra-red sensitive camera while the subject sat relaxed with their head on a chinrest. Ocular torsion was subsequently tracked off-line using iris striations. Each subject separately underwent water caloric irrigation, in accordance with the British Society of Audiology guidelines. For the caloric test, eye movement was recorded in the yaw axis using electro-oculography. For both EVS and calorics, inter-aural response asymmetry was calculated to determine the extent of canal paresis. Both tests revealed impaired vestibular function in the ipsilesional ear of VS patients, with a mean asymmetry ratio of 15 ± 17% and 18 ± 16% for EVS and calorics, respectively. Overall, the caloric test results discriminated controls from patients slightly more effectively than EVS (Cohen's D effect size = 1.44 vs. 1.19). Importantly, there was a significant moderate correlation between the AR values produced by EVS and calorics (*r* = 0.53, *p* < 0.01), and no significant difference between mean AR estimates. When questioned, ≥85% of participants subjectively preferred the EVS experience, in terms of comfort. Moreover, it took ~15 min to complete, vs. ~1 h for caloric. These results confirm that the results of the EVS test broadly agree with those of caloric irrigation, in terms of detecting vestibular asymmetry. Furthermore, they suggest a higher degree of convenience and patient comfort.

## Introduction

Caloric irrigation is currently the most widely used test of vestibular function (1). It is thought to work by inducing convection currents within the endolymph of the horizontal canal [Although, see (2)], evoking nystagmus in the yaw axis. When each ear is stimulated separately, a left-right asymmetry >20–30% in peak slow-phase eye velocity is considered evidence of a vestibular deficit (1). While the caloric test is relatively inexpensive (3), it does have limitations. Firstly, it only assesses lateral canal function (4). Secondly, the ocular response exhibits considerable within- and between-subject variance, presumably due to anatomical variations affecting thermal transfer. Thirdly, it represents a very low frequency physiological motion stimulus [~0.003 Hz; (5)]. This may miss any vestibular deficit occurring at the higher frequency range of movement. Lastly, and perhaps most importantly, caloric irrigation has limitations in terms of patient convenience and applicability (6). The test is time consuming, somewhat inconvenient and has several contraindications. These include excessive ear wax which precludes normal thermal transfer, and abnormal tympanometry, where irrigation may cause pain and/or further eardrum damage (although air calorics may be appropriate in this case) (7). Other exclusion factors include uncontrolled hypertension and mental illness, probably because of the powerful sense of vertigo induced by the stimulus. A 2016 audit within the ENT department of The Queen Elizabeth Hospital Birmingham found that, when complying with The British Society of Audiology guidelines, 33% of patients requiring investigation were unable to undergo the caloric test.

A potential alternative diagnostic test of vestibular function is electrical vestibular stimulation (EVS) (8). EVS involves small cutaneous currents, typically <10 mA, applied to the mastoid processes. This alters the firing rate of the vestibular afferents, leading to postural responses and eye movements. It can be applied either binaurally, with an electrode attached to each mastoid process, or monaurally, with an electrode over one mastoid and a separate reference electrode placed distally, over the spinous C7 process for example. In both cases, the stimulus evokes a sensation of head roll, and this induces concomitant sway and eye movement responses. The eye movement consists primarily of a torsional rotation towards the anode electrode (9). This is accompanied by a slight disconjugate translational motion, whereby the intorting eye elevates and the extorting eye is depressed (10). If the current is maintained, in addition to the torsional offset, torsional nystagmus is observed, with alternating slow and fast movements (11). The ocular response to EVS is primarily due to stimulation of semi-circular canal afferents (8, 12), and resembles the response to head roll motion (13).

To our knowledge, EVS was first applied clinically by Dix et al. (14). They reported attenuated EVS-evoked sway responses following ototoxic damage caused by systemic streptomycin treatment. More recently, EVS-evoked eye movements have been studied in other vestibular pathologies. For example, attenuated responses have been demonstrated following systemic gentamicin treatment (15), while an *increase* in the magnitude of the evoked eye movement has been shown in Ménière's disease (16). Furthermore, specific changes in the 3D kinematics of the eye movement have been related to particular canal deficits (17). These studies suggest that the EVS response is sensitive to both peripheral and central vestibular deficits, and could also help differentiate deficits caused by different canals.

Much of the previous research into the diagnostic potential of the EVS-evoked eye movement has involved the use of scleral coils, which are invasive and impractical for routine clinical use. We recently developed a non-invasive technique using an infrared camera to record the eye movement evoked by a sinuosoidally-varying EVS current (18). This stimulus generates a small yet trackable torsional oscillation of the eye at the same frequency. This response is tracked off-line using iris striations as markers. By applying this technique to patients with vestibular deficits, we recently obtained the proof-of-principle that it can be used to detect vestibular paresis (19). However, given the ubiquity of the caloric test, it is also important to know whether it is equally effective, diagnostically. Here we perform a direct comparison of the EVS test against the caloric irrigation test in patients with vestibular schwannoma. We determine if the extent of canal paresis correlates between the two tests. Furthermore, we compare the convenience and subjective experience of the two tests from the patient perspective.

## Methods

### Participants

Thirty patients with unilateral vestibular schwannoma (VS; 16 male) aged 23 to 75 (mean ± SD; 60 ± 14 years) were recruited from The Centre for Rare Diseases at The Queen Elizabeth Hospital, Birmingham. The presence of VS was confirmed by magnetic resonance imaging and quantified using maximum cerebellopontine angle. In order to determine any effect of tumour size upon vestibular responses, we classified them by size (intracanalicular and cisternal) using the Koos four-point grading system, G1 < 1 cm, G2 1–2 cm, G3 2–3 cm, G4 > 3 cm (20). Most participants were classified as Koos grade 2 (70%), with 27% being classified as grade 1, and 3% as grade 3. No participants were classified as grade 4. The upper limit on tumour size in our cohort is partially attributable to the treatment procedure at Queen Elizabeth Hospital, Birmingham, whereby tumours over 3 cm in diameter are typically treated surgically. Patient tumour measurements and symptoms are presented in Table 1. Thirty healthy controls (16 male) aged 24–82 (mean ± SD; 61 ± 19 years) with no known vestibular disorders were also studied for comparison. The experiment was approved by South Birmingham Research Ethics Committee and performed in accordance with the Declaration of Helsinki. All participants gave informed written consent to participate.

**Table 1:** Patient tumour characteristics and symptoms.

**ID**	**VS side**	**Location**	**Tumour type**	**PTA (dB)**	**CPA (mm)**	**ICL (mm)**	**ICD (mm)**	**Koos grade**	**HL**	**TIN**	**BD**
1	R	IAC	Solid	59		10.2	7.6	I	+	+	+
2	R	IAC	Solid	26		4.6	4	I	+	+	+
3	L	IAC	Solid	48		10	4.1	I	+	+	+
4	R	CPA	Solid	66	9.5	13.3	9.4	II	+	+	–
5	L	CPA	Solid	69	14.6	20.7	12	II	+	–	+
6	L	CPA	Solid	54	18.7	21	14	II	+	+	+
7	L	CPA	Solid	60	7.7	11.4	7.3	II	+	+	–
8	R	CPA	Solid	23	12.5	16.9	9.2	II	+	+	–
9	L	CPA	Cystic	59	14.8	15.4	10.7	II	+	+	+
10	L	IAC	Solid	56		10.6	7.3	I	+	+	+
11	L	CPA	Solid	71	15.7	20.9	14	II	+	+	–
12	R	CPA	Solid	0	12.3	16.4	12.3	II	+	+	+
13	L	IAC	Solid	59		11.9	8.9	I	+	+	+
14	L	CPA	Solid	51	11.9	11.3	11.9	II	+	+	–
15	L	CPA	Solid	71	14.7	12.2	12.2	II	+	+	+
16	R	CPA	Solid	48	11	10.8	8.1	II	+	+	+
17	R	CPA	Solid	63	16	16.6	12	II	+	+	+
18	L	CPA	Solid	0	22	23.1	12.8	III	+	+	+
19	L	CPA	Solid	76	11.4	17.6	11	II	+	–	+
20	R	CPA	Solid	41	10.6	14.4	9.4	II	+	–	+
21	R	CPA	Solid	66	9.9	15.4	9.9	II	+	+	+
22	R	CPA	Solid	53	13.9	16.8	12.1	II	+	+	–
23	L	IAC	Solid	5		8.7	6.2	I	–	+	+
24	L	CPA	Cystic	69	14.6	16	11.3	II	+	+	+
25	L	IAC	Solid	31		4.2	3	I	+	+	+
26	R	IAC	Solid	0		8.6	4.9	I	+	+	–
27	L	CPA	Cystic	45	11.8	10	11.8	II	+	–	–
28	R	CPA	Solid	53	14.6	17.8	13	II	+	+	+
29	L	CPA	Solid	23	5	12	5	II	+	+	+
30	R	CPA	Solid	50	14	13.5	11	II	+	–	–
Mean ± SD				51 ± 17	13 ± 4	14 ± 5	10 ± 3				

*R, right; L, left; IAC, internal auditory canal; CPA, cerebellopontine angle; PTA, pure tone average; SDS, speech discrimination score; ICL, intracanalicular length; ICD, intracanalicular diameter; HL, hearing loss; TIN, tinnitus; BD, balance disturbance; +, symptomatic; –, non-symptomatic*.

### EVS-Evoked Torsional Eye Movements

**Figure 1:**
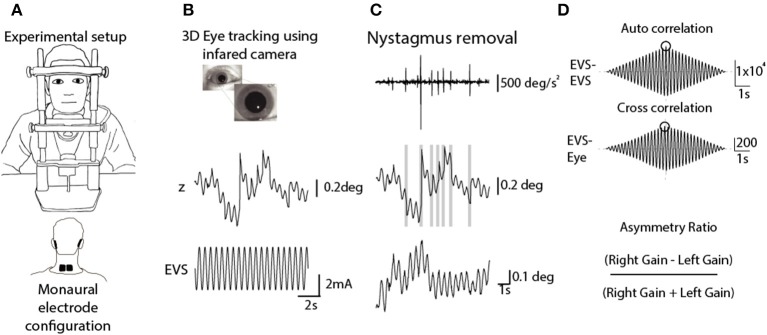
Analysis of EVS-evoked ocular responses. Adapted from Mackenzie and Reynolds (18). **(A)** Subjects sat in darkness with the head fixed during monaural stimulation (10 s, 2 Hz, ±2 mA). **(B)** 3D eye movements were recorded using an infrared camera and then tracked off-line. **(C)** An eye acceleration threshold was used to detect fast phase movements which were then removed using a compensatory inverse nystagmus algorithm. **(D)** Response gain was determined by the ratio of the peak EVE-eye cross correlation to the peak EVE-EVS auto correlation.

We employed the same protocol reported in Mackenzie et al. (19) to measure the ocular response to electrical vestibular stimulation (EVS). Participants were seated with their head resting on a chinrest (SR Research Ltd. Ontario, Canada) for the duration of each 10 s stimulation period (Figure 1A). They were instructed to focus on the lens of an infrared lens camera and instructed to not blink, before being immersed into darkness. The eye was illuminated with invisible infrared light during recording (940 nm), but no visible fixation light was provided.

EVS was delivered via carbon rubber electrodes (46 ×37 mm) smeared with conductive electrolyte (Signa gel, Parker, USA) and attached to the mastoid processes with surgical tape. In order to assess each ear separately, stimulation was applied to one mastoid at a time, with a reference electrode placed over the spinous C7 process. An isolated constant-current stimulator delivered the stimulus (Model 2200, AM Systems, Carlsberg, WA, USA). The stimulus consisted of a 2 Hz sinusoidal waveform, with a peak of ±2 mA, lasting for 10 s. This frequency was chosen since it provides the best compromise between response signal to noise ratio, and patient comfort (18). Although the stimulus could be clearly felt, no subject reported discomfort or pain, and topical anaesthetic was not necessary. Each ear was stimulated 6 times giving a total of 12 trials.

Video images of the eye were sampled at 50 Hz using an infrared-sensitive camera (Grasshopper 3, Point Grey Research Inc., Richmond, BC, Canada). Iris striations were subsequently tracked offline to determine ocular torsion (Figure 1B). This was done using commercially available planar tracking software (Mocha Pro V5, Imagineer Systems Ltd. Guildford, UK). This technique has previously been validated across stimulation frequency range of 0.05–20 Hz (18). Nystagmus fast phases were automatically identified and removed using an inverse nystagmus algorithm (Figure 1C). The magnitude of the eye position response was measured as the peak value of the stimulus-response cross-correlation, using the Matlab XCORR function (units in mA/deg). To normalise this value with respect to the input stimulus, it was divided by the peak of the stimulus autocorrelation (units in mA^2^). This resulted in a measure of response gain which was independent of trial length (units in deg/mA). An asymmetry ratio was then calculated from the gains of both ears (Right Gain—Left Gain/Right Gain + Left Gain) (19) (Figure 1D).

### Caloric Irrigation

Water caloric irrigation was performed with electro-oculography (EOG) according to the procedure recommended by The British Society of Audiology (https://www.thebsa.org.uk/resources/recommended-procedure-caloric-test/). Participants lay supine on an examination bed with their head pitched forward by 30 degrees to bring the horizontal semicircular canals into the vertical plane (21). Irrigations were delivered at 30 ± 0.4 and 44 ± 0.4°C and lasted 30 s during which 250 ± 10 ml of water was delivered (ICS NCA 200 Caloric irrigator, Otometrics, Denmark). Four irrigations were delivered in a Warm Left (WL), Warm Right (WR), Cool Right (CR), Cool Left (CL) stimulus pattern. Participants were instructed to look straight ahead with the eyes open while wearing opaque goggles to prevent visual fixation. Eye movement recording continued for ~3 min after the cessation of irrigation, with 7 min intervals between irrigations (22). During each recording, participants were asked to perform a simple cognitive task to remain alert during the trial (23, 24). This consisted of naming European cities or boys/girls names in alphabetical order.

**Figure 2:**
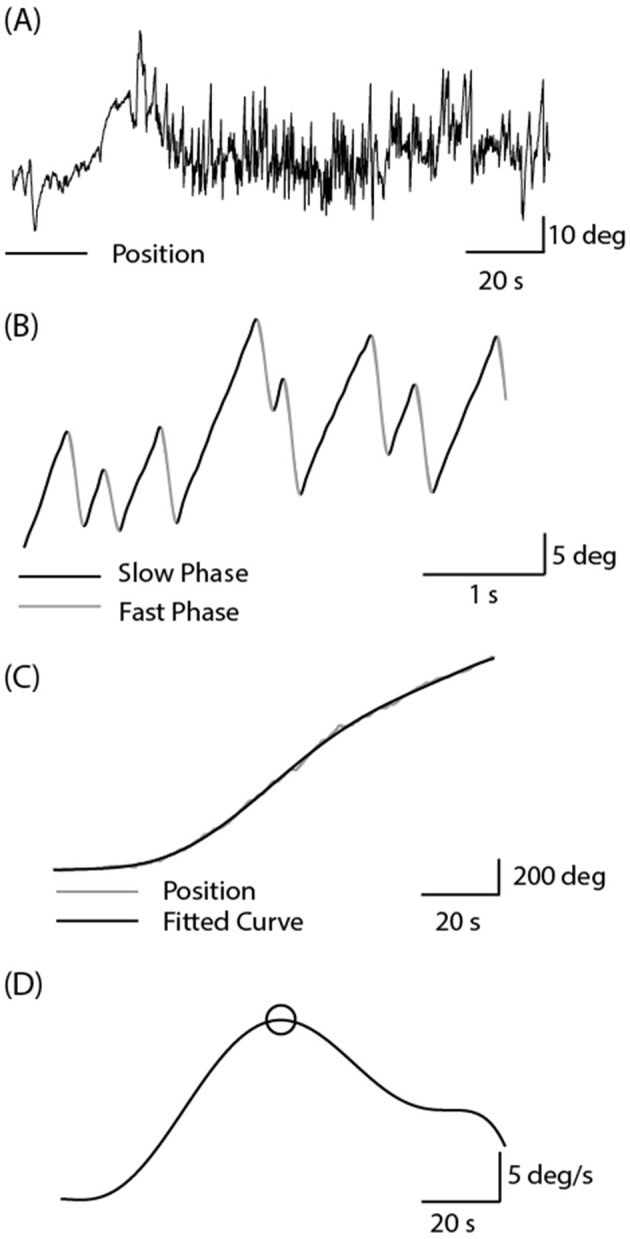
Analysis of caloric-evoked ocular responses. **(A)** Eye position was recorded using EOG in complete darkness following the cessation of the water irrigation. High frequency noise was removed using a Savitzky-Golay filter. **(B)** Slow and fast phase components were identified using the k-means clustering technique in Matlab. **(C)** Slow phase segments were concatenated, and a curve was fitted to this position signal. **(D)** Peak SPV was identified from the differentiated position signal.

Medio-lateral eye position was sampled at 1 kHz using EOG (LP511 amplifier, Grass Technologies). After preparing the skin with Nuprep, two non-polarizable skin electrodes were attached to the outer canthi, with a reference electrode on the forehead. Calibration was achieved by having the participant make saccades towards three visual targets prior to each irrigation (±20 and 0°). Calibrated eye position was smoothed and differentiated using a Savitzky-Golay filter (3rd order, 199 sample frame length) before being down-sampled to 100 Hz (Figure 2A). K-means clustering was applied to the eye velocity signal to identify slow and fast phases of nystagmus (Figure 2B). Slow phase position segments were then concatenated, and a 7th order polynomial was fitted to the resulting data (Figure 2C). The maximum value of this fitted curve was taken as the peak slow phase velocity (SPV) for the trial (Figure 2D). The Jongkees formula (1962) was used to calculate canal paresis from the SPV;

$$\begin{array}{l} \frac{\left(WR\;+\;CR\right)\;-\;\left(WL\;+\;CL\right)}{WR\;+\;WL\;+\;CR\;+\;WL}\;\times 100 \end{array}$$

### Statistical Analysis

Response gain was used to quantify EVS-evoked torsional eye movements, and peak SPV was used to quantify the magnitude of the caloric-evoked response. A paired *t* test (SPSS) was used to compare responses to left and right ear stimulation in control subjects. To ensure that patients “healthy” ears were indeed healthy, they were compared to a random selection of left or right control ears using an independent *t* test. An unpaired *t* test was used to compare asymmetry ratios (AR's) between controls and patients. Effect size (difference in AR between controls and patients) was measured using Cohen's D, using the pooled variance across both groups. Pearson correlation coefficients were used to determine the caloric asymmetry ratio–EVS asymmetry ratio relationship, and tumour size-AR. Bland-Altman analysis was also used to compare tests, due to the limitations of correlation analysis (25). This consisted of plotting the difference between AR estimates for each patient against the mean value for both tests. The resulting plot offers a direct comparison of test values as a function of overall asymmetry values.

For all statistical tests, significance was set at *p* < 0.05. Means and standard deviations are presented in text and figures unless otherwise stated.

## Results

### EVS-Evoked Torsional Eye Movements

**Figure 3:**
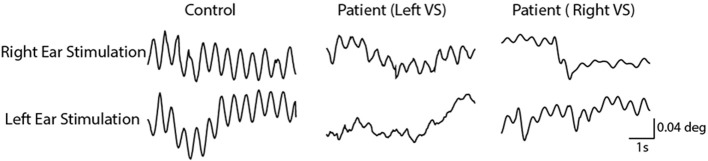
Torsional eye movements evoked by EVS stimulation. EVS induces a sensation of head roll about the naso-occiptal axis, evoking a torsional eye response. A control subject's torsional eye response to right and left ear stimulation were similar in magnitude. However, the vestibular schwannoma patients show a reduced response magnitude during ipsilesional stimulation.

As previously reported, the 2 Hz EVS stimulus generated a small torsional oscillation of the eye at the same frequency, with negligible translation motion (18, 19). Figure 3 depicts EVS-evoked torsional eye position in one control subject and two vestibular schwannoma patients (one left and one right-sided VS), after removal of any fast phases. The control subject's responses were similar in magnitude during both right and left ear stimulation, resulting in an AR of 0.4%. In contrast, both patients showed attenuated responses during ipsilateral stimulation, resulting in AR's of 40 and 26% for left and right sided tumours, respectively.

**Figure 4:**
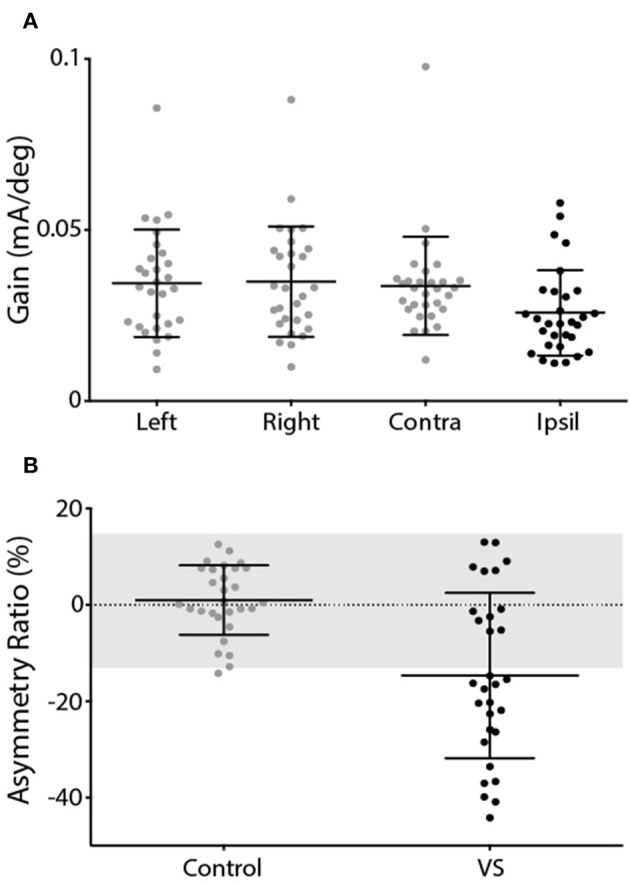
EVS-evoked torsional eye movement response and asymmetry ratio. **(A)** Response gains for controls left and right ear stimulation (grey) and patients contralesional (grey) and ipsilesional ear (black). **(B)** Asymmetry ratio for controls (grey) and patients (black). The grey region depicts the range of a healthy response (±2 SD of control data). Mean and SD presented.

This was reflected by the group data shown in Figure 4. Control subjects showed no difference in response gain for left and right ear simulation [*T*_(28)_ = −0.76, *p* = 0.45]. In patients, stimulation of the contralesional ear produced similar response gains to control subjects [*T*_(55)_ = 0.20, *p* > 0.05], whereas ipsilesional responses were attenuated [*T*_(55)_ = 2.29, *p* < 0.05] (Figure 4A). This was confirmed by a significant difference in AR between controls and patients [Figure 4B; Controls = 0.98 ± 7.2%, Patients = −14.68 ± 17.2%, *T*_(57)_ = 4.54, *p* < 0.001]. The difference in AR between patients and controls corresponds to an effect size of 1.19 (Cohen's D).

### Caloric Irrigation Response

**Figure 5:**
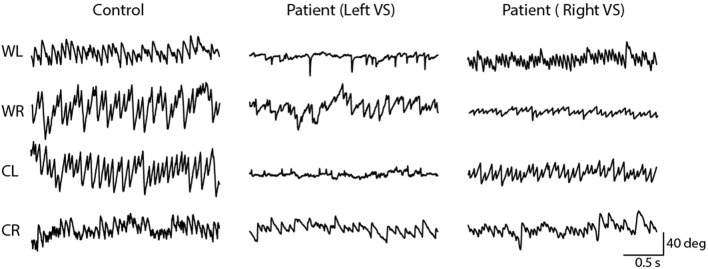
Horizontal nystagmus evoked by caloric stimulation. The direction (down; rightwards, up; leftwards) of the slow phase is dependent on the ear and temperature. A controls subject's responses produced similar slow phase velocity during left and right ear stimulation. However, vestibular schwannoma patients show an attenuated response during ipsilesional stimulation.

Figure 5 depicts horizontal nystagmus evoked by caloric irrigation in one control subject and two schwannoma patients (one left, and one right-sided VS). A clear nystagmus is seen during all four irrigation conditions in the control subject, and also in the contralesional ear of the VS patients. However, ipsilesional irrigation produced a less pronounced nystagmus response. These representative subjects exhibited AR's of 0.7, 26.4, and 33.6% for the control subject, and the left and right sided VS patients, respectively.

**Figure 6:**
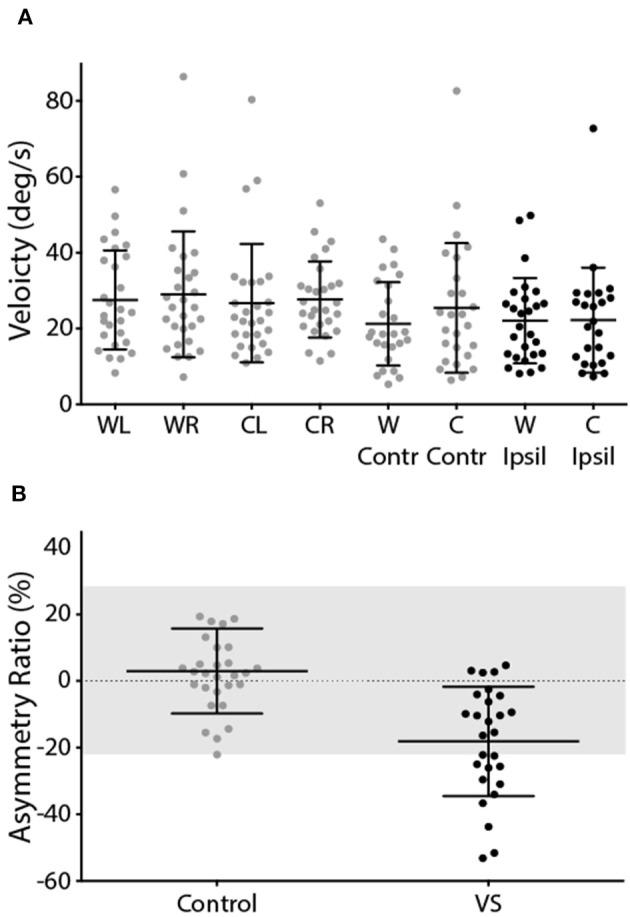
Caloric-evoked peak SPV and asymmetry ratio. **(A)** Peak SPV for controls left and right ears (grey). Patients contralesional (grey) and ipsilesional (black) SPV. **(B)** Asymmetry ratio for controls (grey) and patients (black). The grey region depicts the range of a healthy response (±2 SD of control data). Mean and SD presented.

The mean response to caloric irrigation is shown in Figure 6. There was no significant difference in peak SPV for left and right ear irrigation in control subjects [*T*_(26)_ = −0.16, *p* > 0.05] (Figure 6A). VS patients displayed peak SPV values that were indistinguishable from control subjects during contralesional irrigation [*T*_(56)_ = −0.67, *p* > 0.05]. Ipsilesional responses were significantly attenuated, compared to controls [*T*_(54)_ = 2.68, *p* < 0.05]. The Jongkees formula (26) was used to calculate AR, which was significantly greater in VS patients [Figure 6B; Controls = 3.0 ± 12.7%, Patients −18.1 ± 16.4%; *T*_(54)_ = 5.39, *p* < 0.001]. The difference in AR between patients and controls corresponds to an effect size of 1.44 (Cohen's D).

### EVS-Caloric Comparisons

**Figure 7:**
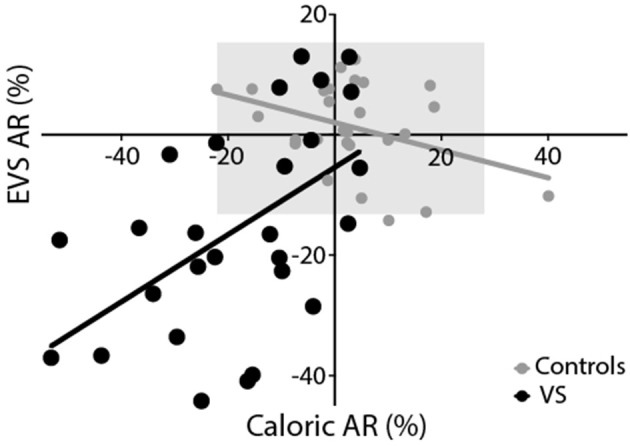
Experimental comparison. Both EVS and caloric tests produced similar asymmetry ratios, resulting in a significant positive correlation for controls (grey) and patients (black). The grey region depicts the range of healthy responses for both tests (±2 SD of control data).

Figure 7 depicts the AR calculated from the caloric reflex and EVS tests plotted against each other. The two methods exhibited a significant correlation for both groups (controls: *r* = −0.39, *p* < 0.05; patients: *r* = 0.53, *p* < 0.01). For caloric irrigation, 37% of patients exhibited AR values beyond 2 standard deviations of the healthy control values. For the EVS response, 60% of patients were outside this range. There was no significant relationship between tumour size and either caloric or EVS AR (*p* > 0.05). This was also true of the pure tone average scores in patients (*p* > 0.05). When explicitly asked to say which of the two tests they preferred in terms of comfort and practicality, the vast majority of volunteers reported a preference for the EVS test over caloric irrigation (Controls: 95%; Patients: 85%).

**Figure 8:**
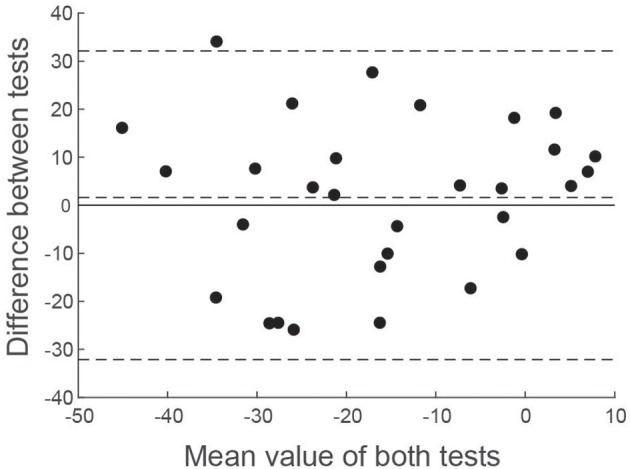
Bland-Altman comparison of EVS and Caloric asymmetry estimates. The difference between VS asymmetry ratios has been plotted against the mean value of both tests. Horizontal dashed lines show the mean value of the difference along with ± 1.96 standard deviations.

Since correlation analysis can be potentially misleading for comparing test performance, we also performed a Bland-Altman analysis (25). Figure 8 shows the difference in asymmetry estimates between tests for each patient, plotted against the mean value across both tests. A number of statistical conclusions can be drawn from this analysis. Firstly, the difference values were normally distributed (Shapiro-Wilk value = 0.96; *p* = 0.39), with all but one value lying within 1.96 standard deviations of the mean. Secondly, there was no difference between tests, in terms of bias (mean difference = 1.6%; *t* = 0.54; *p* = 0.59). Furthermore, there was no significant correlation between difference values and mean values (Figure 8), confirming that test performance was similar across all values of asymmetry.

## Discussion

Previous research has established the diagnostic potential of EVS-evoked torsional eye movement recordings for detecting vestibular paresis (10, 13, 17–19). Here we extended this research to make a direct comparison to caloric irrigation. The results show that EVS compares favourably to calorics for assessing vestibular asymmetry in patients with vestibular schwannoma (VS). Both tests revealed attenuated responses in the ipsilesional ear, and significantly greater asymmetry than seen in age-matched control subjects. A moderate correlation between the two tests (*r* = 0.53) suggests that the result of the EVS test broadly agrees with that of caloric irrigation.

In healthy individuals, the EVS test produced virtually no asymmetry between the ears, as expected. The contralesional (unaffected) ear of VS patients produced similar response gain to the control subjects, whereas their ipsilesional responses were impaired. This resulted in a mean patient AR of ~15%. We previously reported a similar AR in VS (~20%) using the same technique (19). Others have reported values as high as 50% when measuring EVS-evoked eye movements in VS (27). Furthermore, when measuring *sway* responses in VS patients, Welgampola et al. (28) reported an AR of ~40%, and found no overlap between patients and controls, in contrast to our findings. These discrepancies can be explained by considerable differences in tumour size (27 mm in Welgampola et al. vs. 13 mm here), patient numbers (4 patients in Aw et al. vs. 30 here) and, perhaps, methodological differences (e.g., use of scleral coils in Aw et al., vs. video recording here). Nevertheless, we did observe considerable overlap between patient and control data (Figures 4B, 6B). But it is important to emphasise that our aim was not to develop a diagnostic test for VS, a task currently well served by MRI (29). Rather, our intention was to determine whether EVS is comparable to calorics in identifying vestibular asymmetry. Since VS is known to cause vestibular deficits, this pathology provides a useful basis for this comparison. But it is inappropriate to measure “sensitivity” or “specificity” for either test as a diagnostic for VS, largely because some of these patients have mild or absent vestibular deficits despite an obvious tumour seen with MRI. This is apparent in Figures 4B, 6B, where the patient group exhibits considerable overlap with control subjects, for both EVS and calorics. So, the relevant point is not absolute test performance *per se*, but the comparison between the two tests.

Direct numerical comparisons confirm similar performance between tests. In the healthy control group, the EVS test exhibited slightly less asymmetry and variability, compared to calorics [AR's: 1 ± 7% (EVS) vs. 3 ± 13% (Caloric)]. However, in the patient group calorics revealed a greater deficit [AR's: 14.7 ± 17.1% (EVS) vs. 18.1 ± 16.4% (Caloric)]. Overall, the caloric test discriminated patients from controls slightly more than EVS, as reflected by a larger effect size [Cohen's D = 1.19 (EVS) vs. 1.44 (Caloric)]. The Bland-Altman plot confirmed equitable test performance, with no significant bias of either test over the other in terms of asymmetry (Figure 8). Neither test was found to correlate with tumour size or pure tone average scores and had no relationship with years diagnosed, or whether the tumour was intracanalicular or had a cisternal component. Importantly, the vast majority of patients (85%) and control subjects (95%) subjectively preferred the EVS experience over calorics. Furthermore, in terms of clinical practicality, EVS was faster (EVS, ~15 min; caloric, ~1 h) and more convenient (6).

The moderate correlation we observed between tests corroborates EVS as a measure of canal paresis. Nevertheless, an *r* value of 0.53 equates to an *r*-squared value of 0.28, which implies that 72% of the variance between tests is unexplained. What could account for the difference between tests? Several prosaic explanations exist. For example, some degree of measurement noise is inevitable for both EOG and video tracking. Furthermore, mental arousal is known to affect the magnitude of caloric nystagmus (23). Although our subjects performed a concurrent mental task, it is impossible to control perfectly for arousal. There are other explanations, however, which are of greater physiological and clinical interest. For example, caloric irrigation only tests the integrity of the horizontal canal (4). Since EVS alters activity in all vestibular afferents, it assesses the function of all canals simultaneously (8). Indeed, case evidence suggests that deficits specific to individual canals cause corresponding changes in the 3D kinematics of the EVS-evoked eye movement (17). The superior branch of the vestibular nerve innervates the horizontal and anterior canals. Therefore, any tumour arising from this branch would be expected to produce both an abnormal caloric reflex *and* EVS response. However, 85–91% of VS tumours arise from the inferior branch (30, 31). Isolated damage to this branch might therefore leave the caloric response intact, while affecting the EVS response. Hence, it is conceivable that a deficit restricted to the anterior or posterior canals may not affect the caloric response, but would change the amplitude and kinematic profile of the EVS response. The two tests also differ in terms of the motion stimulus they represent. Caloric irrigation provides an extremely low frequency stimulus, approximately 0.003 Hz (1, 5), whereas the EVS stimulus we applied was 2 Hz. These frequencies likely assess different functional aspects of the vestibular-ocular reflex, and may be differentially susceptible to certain nerve damage. In summary, all of these factors could act to reduce the magnitude of the inter-test correlation coefficient. In control subjects we actually observed a small but significant *inverse* correlation between the two tests (*r* = −0.39). Again, this raises the possibility that both tests may be measuring subtly different aspects of vestibular function. Dissociations have previously been reported between the caloric and head impulse test (32, 33). Hence, the concept of a “gold standard” vestibular test may be inappropriate, since each test has specific advantages and disadvantages, which may render them more or less suitable for revealing particular pathologies. Nevertheless, EVS and caloric results did exhibit a moderate correlation, confirming that they broadly agree in terms of the extent of canal paresis.

As discussed in Mackenzie et al. (19), the diagnostic utility of EVS across a broader range of vestibular disorders may depend upon its precise site of action. EVS currents most likely alter neural firing rate via the spike trigger zone of the primary afferent (8, 34, 35), implying that the response will only reveal deficits downstream of the hair cell. Vestibular schwannoma certainly constitutes such a deficit, which explains the impaired responses seen here. However, it has also been reported that gentamicin-induced vestibular toxicity impairs EVS-evoked eye movements (15). Since acute gentamicin toxicity kills vestibular hair cells, this could be interpreted as evidence that EVS stimulates the hair cell rather than the primary afferent. However, vestibular afferents have a high resting firing rate, and loss of hair cell input may conceivably reduce their firing rate and/or their excitability. Such a loss of excitability could diminish the response to an externally applied current. Irrespective of the precise mechanism of action, the evidence of gentamicin-induced deficits in the EVS-evoked response provides encouraging evidence that it could diagnose peripheral as well as central vestibular deficits (17), at least if such deficits affect hair cell function.

As mentioned above, the response to EVS is thought to be the net effect of simultaneous activation of all semicircular canal afferents (8, 12). The resulting rotation vector is one of head roll, resulting in a predominantly torsional eye movement. This should mean that a vestibular deficit restricted to a specific canal will manifest as a change in the evoked eye movement trajectory, and there is evidence to support this (17). Nevertheless, one limitation of EVS is that individual canals cannot be stimulated separately. This is possible with the head impulse test (HIT), where head movement is tailored to activate unique left-right canal pairings (e.g., both horizontals together, or left superior/right posterior). However, we previously compared the HIT test to EVS in VS patients and found that it was far less effective for revealing vestibular asymmetry (19). This suggests that EVS is a more sensitive detector of asymmetry, at least for VS [see discussion of (19) for more detailed coverage of this issue]. Whether this is true of other vestibular disorders remains to be seen. Another limitation of the EVS response is that it does not assess otolith function, a role currently fulfilled by VEMPs (36).

Diagnostic and physiological considerations aside, EVS offers considerable practical advantages over calorics. A number of parameters need to be considered when performing the caloric test: temperature (±1°C), duration (30 s), flow rate (500 ml ± 10 ml/min), head position (30°C) and patient alertness. Differences in these factors will alter heat transfer to the horizontal canal, altering the magnitude of the evoked nystagmus. A correct head position is important to facilitate the movement of endolymph (21). However, anatomical variation of canal orientation can introduce uncertainty to this alignment. Low patient alertness levels have been shown to yield significantly reduced nystagmus output (23) during caloric stimulation. Similar effects may also be true during electrical stimulation; however, this is yet to be determined. If a caloric is unsuccessful and needs to be performed again, habituation becomes a consideration. EVS on the other hand requires very little skill during patient preparation and testing. Electrode placement over the mastoid processes is an element of consideration to ensure sufficient current transfer, after which a computer can run the entire examination with little experimenter involvement. The invasive nature of the caloric reflex test can be uncomfortable for some patients and also carries a small risk of accidentally ear canal trauma, due to the absence of a fat pad under the skin in the proximal regions, as well as risks of infection. Fluid that enters the ear has to be sterile but any fluid which remains in the ear following irrigation can facilitate the growth of pathogens. Acute otitis externa is the most common infection that can develop following a caloric reflex test (37). EVS can be uncomfortable if the frequency or intensity is high, but we found that our 2 Hz ± 2 mA stimulus was well tolerated. Careful inspection of the skin over the mastoid is needed to ensure no abrasions are present, as the resulting low impedance might focus the current through this area, leading to discomfort. The time it takes to perform each test is vastly different with a caloric reflex test taking up to 1 h while an EVS tests can be performed in under 15 min.

In summary, we have demonstrated that EVS-evoked eye movements can be use used to detect vestibular asymmetry in patients with vestibular schwannoma, and that the level of asymmetry correlates with that measured by the caloric test. EVS may be more suitable for clinical use as it is quick, more comfortable for participants and tests all canals. Further work is required to determine its diagnostic potential in wider group of pathologies, but previous research strongly suggests it is can be applied to both central and peripheral vestibular deficits (14, 15).

## Data Availability Statement

The datasets generated for this study are available on request to the corresponding author.

## Ethics Statement

The studies involving human participants were reviewed and approved by South Birmingham Research Ethics Committee. The patients/participants provided their written informed consent to participate in this study.

## Author Contributions

SM and RR: study design. SM, RI, PM, and AD: participant recruitment. SM: data collection. ND: MRI measurements. SM: data analysis. SM and RR: manuscript preparation. KL: training in caloric technique.

### Conflict of Interest

The authors declare that the research was conducted in the absence of any commercial or financial relationships that could be construed as a potential conflict of interest.
